# Non-Invasive Physical Plasma Generated by a Medical Argon Plasma Device Induces the Expression of Regenerative Factors in Human Gingival Keratinocytes, Fibroblasts, and Tissue Biopsies

**DOI:** 10.3390/biomedicines10040889

**Published:** 2022-04-13

**Authors:** Benedikt Eggers, Matthias Bernhard Stope, Jana Marciniak, Werner Götz, Alexander Mustea, James Deschner, Marjan Nokhbehsaim, Franz-Josef Kramer

**Affiliations:** 1 Department of Oral, Maxillofacial and Plastic Surgery, University Hospital Bonn, 53111 Bonn, Germany; franz-josef.kramer@ukbonn.de; 2 Department of Gynecology and Gynecological Oncology, University Hospital Bonn, 53127 Bonn, Germany; matthias.stope@ukbonn.de (M.B.S.); alexander.mustea@ukbonn.de (A.M.); 3 Department of Orthodontics, University Hospital Bonn, 53111 Bonn, Germany; jana.marciniak@ukbonn.de (J.M.); wgoetz@uni-bonn.de (W.G.); 4 Department of Periodontology and Operative Dentistry, University Medical Center of the Johannes Gutenberg University, 55131 Mainz, Germany; james.deschner@uni-mainz.de; 5 Section of Experimental Dento-Maxillo-Facial Medicine, University Hospital Bonn, 53111 Bonn, Germany; m.saim@uni-bonn.de

**Keywords:** non-invasive physical plasma, cold atmospheric plasma, cold atmospheric pressure plasma, gingival keratinocytes, gingival fibroblasts, tissue biopsies, wound healing, in vitro

## Abstract

After oral surgery, intraoral wound healing and tissue regeneration is an important factor for the success of the entire therapy. In recent years, non-invasive medical plasma (NIPP) has been shown to accelerate wound healing, which would be particularly beneficial for patients with wound healing disorders. Since the application of NIPP in dentistry has not been sufficiently understood, the aim of the present study was to investigate the effect of a medical argon plasma device on gingival cells. Human gingival fibroblasts, keratinocytes, and tissue biopsies were treated with NIPP for different durations. Crucial markers associated with wound healing were examined at the mRNA and protein levels by real-time PCR, ELISA and immunohistochemistry. NIPP treatment led to an increase in Ki67 and MMP1 at mRNA and protein levels. NIPP application lasting longer than 60 s resulted in an increase in apoptotic genes at mRNA level and superficial damage to the epithelium in the tissue biopsies. Overall, our experimental setup demonstrated that NIPP application times of 30 s were most suitable for the treatment of gingival cells and tissue biopsies. Our study provides evidence for potential use of NIPP in dentistry, which would be a promising treatment option for oral surgery.

## 1. Introduction

In dentistry, especially in the field of oral and maxillofacial surgery, intraoral wound healing is a key factor for the success of an intervention. In particular, the regeneration of the soft tissue is essential for the underlying structures of the bone, as this is an important barrier against microorganisms, a large number of which reside in the oral cavity [1]. In addition, the gingiva is a barrier against antigens found in the air, which make their first contact with human organisms there before passing into the gastrointestinal and respiratory tracts [2].

The gingiva consists of a surface epithelium separated from the underlying dense connective tissue by a basal lamina [3]. The surface epithelium is a multilayered squamous epithelium that is keratinised in the masticatory areas and nonkeratinised in the other lining areas. For this reason, keratinocytes and fibroblasts are predominantly found in this tissue [3]. An important element of the connective tissue is collagen 1: Its main structural component collagen type 1 α1 (COL1A1) is produced by fibroblasts and can also be influenced by keratinocytes. [4,5].

The interaction of various factors contributes to wound healing: Various cytokines are involved not only in inflammatory responses, but also in critical functions of wound healing such as wound contraction or tissue remodeling [6,7,8,9,10]. During wound healing, the reorganization of collagen is essential for the entire tissue repair [11]. This process of remodeling is mainly controlled by matrix metalloproteinases (MMP)s, such as MMP1 [12]. The main characteristic of a healing wound is a high proliferation rate of dividing cells, as indicated by high levels of proliferation-associated proteins Ki67 and Proliferating Cell Nuclear Antigen (PCNA) [13,14]. Ki67 is found in all active cell cycle phases, but it is not present in quiescent cells [15]. PCNA is a nuclear protein that is obligatory for DNA synthesis and is mainly expressed during the longer G1 and S phases of the cell cycle [16].

Within the last 10 years, non-invasive physical plasma (NIPP) has become the focus of medical research. NIPP is a highly reactive, electrically conductive gas, also known as the 4th state of matter, which has various properties [17]. For example, antimicrobial, antioncogenic, and wound-healing effects have been described for the medical field [18,19,20,21,22,23]. Several types of NIPP devices have been designed: Plasma jets, Dielectric Barrier Discharges (DBD), and hybrid devices that combine both technologies [24]. In all devices, plasma is generated between the cathode and anode by a high-frequency voltage. In DBD devices, the treated object is the counter-electrode, whereas in plasma jets, a carrier gas is used to deliver NIPP from inside the device to the target [25]. Various clinical studies have also shown a supportive effect of different NIPP devices on gingival wound healing [26,27]. However, a more in-depth molecular investigation of the effect of NIPP on gingival cells and tissues has not yet been described. The aim of the study was therefore to investigate the effect of NIPP generated by a medical argon plasma device on human gingival keratinocytes (HGK) and human gingival fibroblasts (HGF) regarding crucial molecules involved in wound healing. Since apoptotic effects by NIPP have also been described [19], the effects on crucial apoptotic molecules, such as caspase (CASP)9 and CASP3, were also to be investigated in this study. In addition, the effect of NIPP should also be demonstrated histologically in tissue biopsies of gingival tissue to demonstrate the effect of a regular clinical use of NIPP in dentistry.

## 2. Materials and Methods

### 2.1. Cell Culture

HGF (HGF-1; CRL-2014) and HGK (Primary Gingival Keratinocytes; PCS-200-014) were obtained from ATCC (Manassas, VA, USA). HGF were propagated in Dulbecco’s modified essential medium (DMEM; Invitrogen, Waltham, MA, USA), containing 10% fetal bovine serum (FBS; Invitrogen, Waltham, MA, USA), and 1% penicillin/streptomycin (Invitrogen) at 37 °C with 5% CO_2_ and 95% humidity. The cultivation of HGK was performed under the same conditions in Keratinocyte Growth Medium 2 (PromoCell, Heidelberg, Germany), supplemented with 1% penicillin/streptomycin. The cell culture medium was changed every 2–3 days. For the experiments, 50,000 cells/well were each cultivated in 3.5 cm dishes (VWR, Radnor, PA, USA). One day prior to the experiments, FBS concentration of HGF was reduced to 1%.

### 2.2. NIPP Application

NIPP was generated by an argon plasma jet (kINPen med, neoplas med, Greifswald, Germany) at 4.0 L of argon per minute. The treatment of medium covered cells was performed at a distance of 2 cm (nozzle to cells), describing a spiral for the indicated times.

### 2.3. Analysis of mRNA Expression

One day after the experiments, the RNA was extracted using a RNeasy Kit (Qiagen, Hilden, Germany) according to the manufacturer’s instructions. An iScript™ Select cDNA Synthesis Kit (Bio-Rad Laboratories, Munich, Germany) was used to reverse transcribe 1 µg RNA into cDNA. A mixture of 12.5 µL SsoAdvanced™ Universal SYBR^®^ Green Supermix (Bio-Rad), 2.5 µL commercially available primer (*GAPDH*, *Ki67*, *PCNA*, *COL1A1*, *MMP1*, *CASP9*, and *CASP3*; QuantiTect Primer Assay, Qiagen), and 9 µL deionised water were used to amplify 1 µL cDNA by the iCycler iQ™5 detection system (Bio-Rad). For real time PCR, the following protocol was used: 5 min heating at 95 °C, 40 cycles denaturation at 95 °C for 10 s and combined anealing/extension at 60 °C for 30 s. The data were evaluated using the comparative threshold cycle method.

### 2.4. Analysis of Ki67 and MMP1 Protein Levels

Ki67 protein levels were analysed in cell lysates using the Human Ki67 enzyme-linked immunoassay (ELISA) Kit (ab253221, abcam, Cambridge, UK). For quantification of MMP1 protein levels, cell supernatants were analysed using the Human Total MMP-1 DuoSet (ELISA) kit (DY901, Bio-Techne, Minneapolis, MI, USA) according to the manufacturer’s instructions. Absorbance was measured at 450 nm using a microplate reader (Epoch™ Microplate Spectrophotometer, BioTek Instruments, Winooski, VT, USA) and normalized to total protein concentration using Pierce BCA Protein Assay Kit (23227, Thermo Scientific, Pierce Biotechnology, Rockford, WA, USA). The absorbance was measured at 570 nm, as described above.

### 2.5. Caspase-3/7 Assay

Cells were seeded into 3.5 cm dishes, as described above, and were subsequently treated with NIPP. Immediately after treatment, cell suspension was transferred to 96 well plates and incubated for 24 h and 48 h. After incubation, medium was removed and 100 μL of Caspase 3/7 detection solution (CellEventTM Caspase 3/7 Green Detection Reagent, Thermo Fisher Scientific, Waltham, MA, USA) was added according to the manufacturer’s instructions for 45 min. Staurosporine (Sigma-Aldrich, St. Louis, MO, USA) treated cells served as positive control. Fluorescens was measured at 535 nm following excitation at 495 nm using a microplate reader (Tecan, Männedorf, Switzerland).

### 2.6. Staining of Tissue Biopsies

Human gingival biopsies, intraoperatively classified as tissue waste, were taken from five healthy patients during routine surgeries in the Department of Oral Surgery at Bonn University Hospital. The approval of the ethics committee of the University of Bonn (#111/17) and the written informed consent of the patients were obtained in advance. Tissue was rinsed with Phosphate buffered saline (PBS; Invitrogen) and cut into thirds. Two thirds were treated with NIPP using the spacer provided by the company; the other third served as a control group. The tissues were stored in 3.5 cm wells (VWR) in DMEM (Invitrogen), containing 1% FBS (Invitrogen), and 1% penicillin/streptomycin (Invitrogen) at 37 °C with 5% CO_2_ and 95% humidity for 24 h. Subsequently, the pieces of tissues were fixed in 4% paraformaldehyde (Sigma-Aldrich) for 2 d, hydrated, dehydrated in an ascending ethanol series (AppliChem, Darmstadt, Germany), and embedded in paraffin (McCormick Scientific, Richmond, IL, USA). The tissue was sliced into sections of 4 µm and mounted on glass slides (SuperFrost Plus; Thermo Fisher Scientific).

For histological analysis, sections were stained with hematoxylin and eosin (HE; Merck, Darmstadt, Germany), dehydrated, and mounted with DePeX (SERVA Electrophoresis GmbH, Heidelberg, Germany).

For immunohistochemistry, sections were deparaffinated, rehydrated, and rinsed with TBST (Merck) for 10 min. Afterwards, the endogenous peroxidase was blocked by using 0.3% methanol (AppliChem)/H_2_O_2_ (Merck) solution for 10 min. After another rinsing with TBST, the sections were blocked with 5% Bovine Serum Albumin Fraction V (BSA; Roche Diagnostics, Mannheim, Germany) in PBS for 30 min and incubated with primary antibodies rabbit anti-Ki67 (1:2000; abcam), or rabbit anti-MMP1 (1:600; abcam) in 1% BSA in a humid chamber at 4 °C overnight. Following this, the sections were rinsed with TBST and incubated with goat anti-rabbit IgG-HRP secondary antibody (Dako, Glostrup, Denmark) at room temperature for 30 min. The staining was visualized with 3.3′-diaminobenzidine chromogen (Thermo Fisher Scientific) and—after rinsing with TBST—counterstained with Mayer’s hematoxylin (Merck) for 5 s. After the rinsing and dehydration, the slides were mounted with DePeX. Analysis was performed using the Axioskop 2 microscope (Carl Zeiss, Jena, Germany) with an AxioCam MRc camera and the AxioVision 4.7 software (Carl Zeiss).

### 2.7. Statistical Analysis

For statistics, GraphPad Prism Software Version 7 (GraphPad Software, Inc., La Jolla, CA, USA) was used with one-way ANOVA and post hoc Dunnett’s multiple comparisons test. *p* values below 0.05 were defined as statistically significant.

## 3. Results

### 3.1. Analysis of Ki67 and PCNA

**Figure 1:**
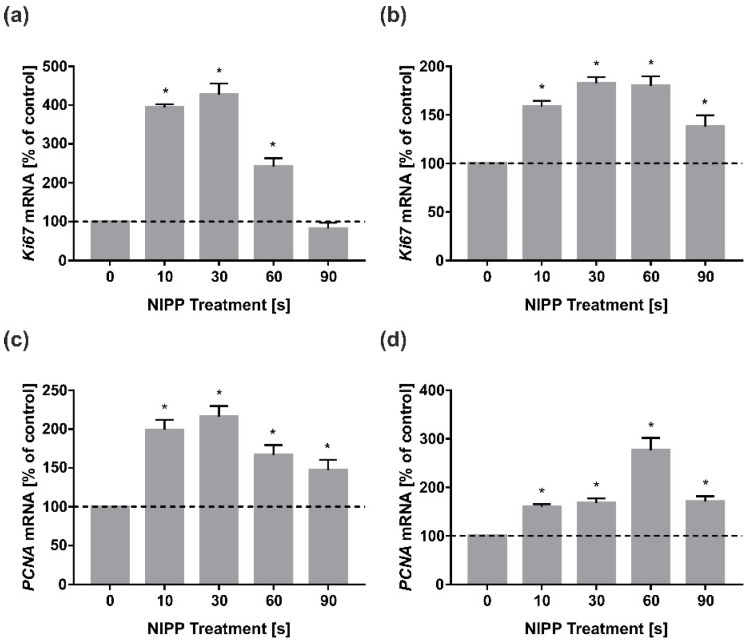
Influence of NIPP on *Ki67* and *PCNA* mRNA levels in HGF and HGK at 24 h. Treatment was performed for the indicated times: (**a**) *Ki67* mRNA in HGF; (**b**) *Ki67* mRNA in HGK; (**c**) *PCNA* mRNA in HGF; (**d**) *PCNA* mRNA in HGK. *n* = 9. * statistical significance compared to control (0 s) (*p* < 0.05).

At first, we investigated the effect of NIPP on cellular growth. In HGF and HGK, treatment with NIPP caused an increase in *Ki67* mRNA levels. In HGF, there was a dose-dependent increase of *Ki67* mRNA for exposure times of 10 s to 30 s (Figure 1a). Longer treatment times resulted in a decrease in *Ki67* mRNA regulation. In HGK, the dose-dependent mRNA upregulation was evident up to an application time of nearly 60 s (Figure 1b). The mRNA regulation of *PCNA* in HGF showed a similar pattern to *Ki67* with a peak at 30 s (Figure 1c). Interestingly, in HGK, the *PCNA* expression maximum also occurred after 60 s of NIPP but was more pronounced than the *Ki67* expression (Figure 1d).

Following the investigation of mRNA expression, we then focused on the examination of protein expression of Ki67.

**Figure 2:**
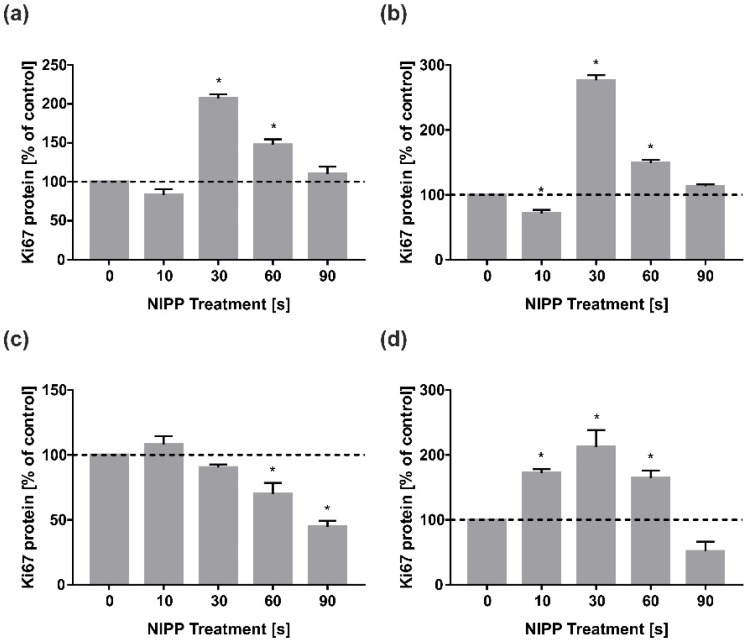
Effects of NIPP on Ki67 protein levels in HGF and HGK at 24 h and 48 h, respectively. Protein levels were related to total protein and normalised to control. Treatment was performed for the indicated times: (**a**) Ki67 concentration in HGF cell lysates at 24 h; (**b**) Ki67 concentration in HGF cell lysates at 48 h; (**c**) Ki67 concentration in HGK cell lysates at 24 h; (**d**) Ki67 concentration in HGK cell lysates at 48 h. *n* = 6. * statistical significance compared to control (0 s) (*p* < 0.05).

In HGF protein expression of both one day and two days was similar to mRNA level at one day (Figure 2a,b). Interestingly, in contrast to mRNA data, 10 s of NIPP treatment showed a significant reduction in Ki67 protein expression after 48 h. However, the protein data confirmed that 30 s was the most effective NIPP treatment time in HGF.

In contrast, in HGK, Ki67 protein levels decreased in a dose-dependent manner after 24 h of incubation, indicating anti-proliferative effects of NIPP (Figure 2c). Interestingly, Ki67 protein expression after 48 h correlated with mRNA data from the 24 h incubation (Figure 1b), demonstrating a dose-dependent stimulatory effect with a maximum at 30 s NIPP treatment (Figure 2d). Similar to the 24 h incubation data in HGK, 90 s NIPP led to a strong reduction of Ki67 protein levels by almost 50% at 48 h (Figure 2d).

### 3.2. Analysis of COL1A1 and MMP1

**Figure 3:**
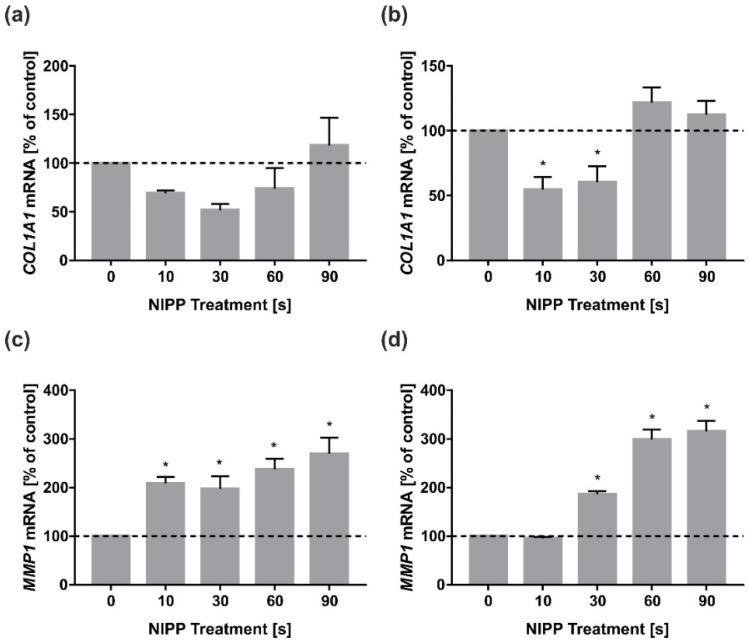
Impact of NIPP on *COL1A1* and *MMP1* mRNA levels in HGF and HGK at 24 h. Treatment was performed for the indicated times: (**a**) *COL1A1* mRNA in HGF; (**b**) *COL1A1* mRNA in HGK; (**c**) *MMP1* mRNA in HGF; (**d**) *MMP1* mRNA in HGK. *n* = 9. * statistical significance compared to control (0 s) (*p* < 0.05).

Next, we focused on the study of marker molecules related to tissue remodeling. First, we examined the mRNA expression of *COL1A1* as a major component of gingival connective tissue. Interestingly, NIPP had little effect on mRNA expression in HGF after 24 h (Figure 3a). In HGK, 10 s of NIPP caused a reduction in *COL1A1* mRNA expression after 24 h—however, only very limited effects of NIPP were observed in HGK (Figure 3b).

In addition, we investigated the influence of NIPP on *MMP1*, which plays a pivotal role in collagen degradation and remodeling.

In HGF, there was a significant increase in *MMP1* regulation in relation to increasing NIPP treatment time (Figure 3c). In HGK, a similar effect was seen, but an increase in *MMP1* mRNA regulation was only seen from 30 s NIPP treatment after 24 h (Figure 3d).

The NIPP effect on the modulation of MMP1 expression was also detected at the protein level.

**Figure 4:**
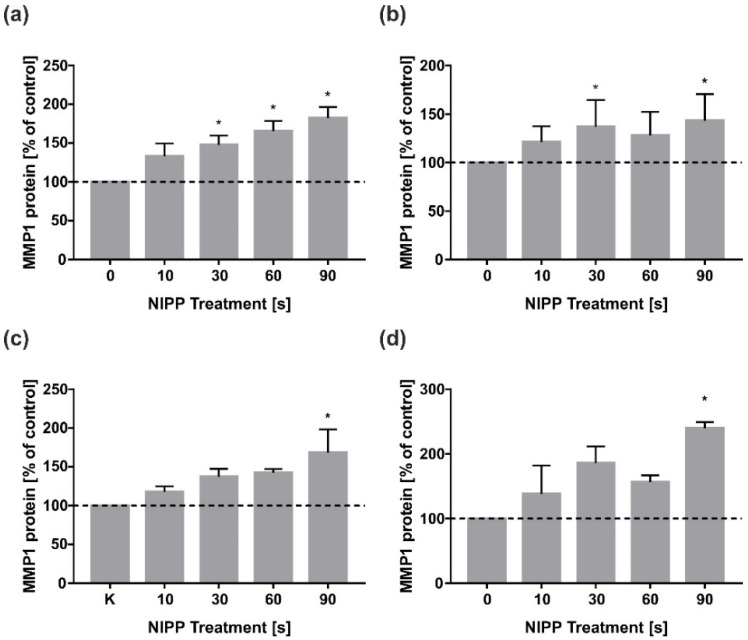
Effects of NIPP on MMP1 protein levels in HGF and HGK at 24 h and 48 h, respectively. Protein levels were related to total protein and normalised to control. Treatment was performed for the indicated times: (**a**) MMP1 concentration in HGF cell culture supernatants at 24 h; (**b**) MMP1 concentration in HGF cell culture supernatants at 48 h; (**c**) MMP1 concentration in HGK cell culture supernatants at 24 h; (**d**) MMP1 concentration in HGK cell culture supernatants at 48 h. *n* = 6. * statistical significance compared to control (0 s) (*p* < 0.05).

In HGF, the dose-dependent effect of the NIPP application time was already apparent after 24 h (Figure 4a) and was also still visible after two days, with a peak at 90 s of NIPP (Figure 4b). HGK showed a similar pattern, with 90 s NIPP also having the strongest effect on MMP1 protein levels at one and two days (Figure 4c,d).

### 3.3. Examination of CASP9 and CASP3

**Figure 5:**
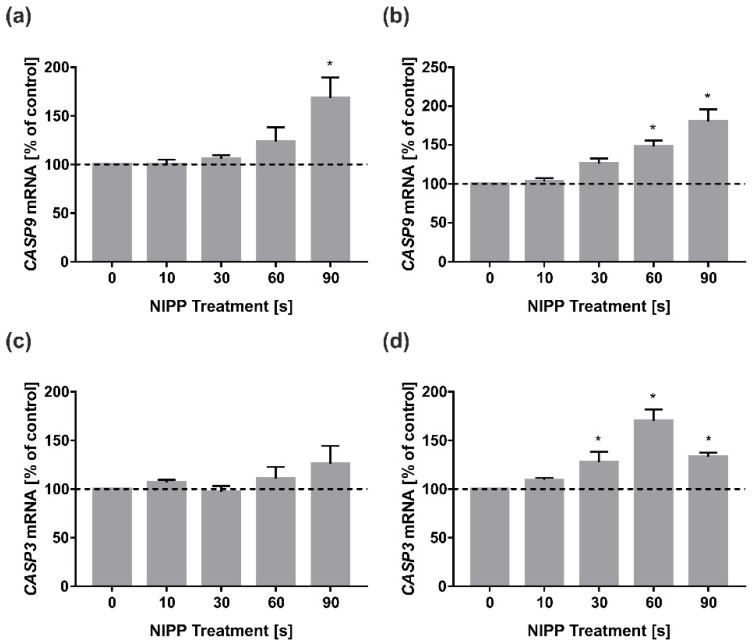
Influence of NIPP on *CASP9* and *CASP3* mRNA levels in HGF and HGK at 24 h. Treatment was performed for the indicated times: (**a**) *CASP9* mRNA in HGF; (**b**) *CASP9* mRNA in HGK; (**c**) *CASP3* mRNA in HGF; (**d**) *CASP3* mRNA in HGK. *n* = 9. * statistical significance compared to control (0 s) (*p* < 0.05).

Finally, we investigated the influence of NIPP on apoptotic genes, as NIPP is also known for its apoptotic effect in cancer cells. It was shown for application times of 90 s NIPP in HGF and 60 s NIPP, as well as 90 s NIPP in HGK, that there was an upregulation in the mRNA expression of *CASP9* (Figure 5a,b). Interestingly, this effect could not be detected with HGF in the mRNA expression of *CASP3*, which is regulating the end of the apoptotic cascade (Figure 5c). In comparison, *CASP3* mRNA was upregulated in HGK at application times of 60 s. Interestingly, the effect of 90 s NIPP was significantly lower on *CASP3* expression (Figure 5d).

**Figure 6:**
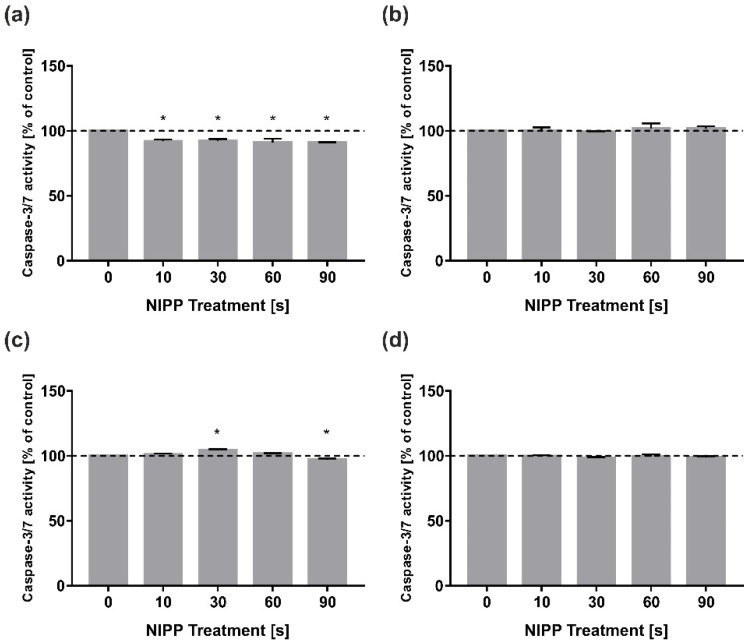
Effects of NIPP on Caspase-3/7 activity in HGF and HGK 24 h and 48 h after treatment, respectively. Data was normalised to control. Treatment was performed for the indicated times: (**a**) Caspase-3/7 activity in HGF at 24 h; (**b**) Caspase-3/7 activity in HGF at 48 h; (**c**) Caspase-3/7 activity in HGK at 24 h; (**d**) Caspase-3/7 activity in HGK at 48 h. *n* = 6. * statistical significance compared to control (0 s) (*p* < 0.05).

The apoptotic effect of NIPP on the cells was measured with a caspase-3/7 assay. Surprisingly, the slight apoptotic effect of NIPP on the cells could not be confirmed: Both HGF and HGK showed no significant increase of active caspase 3/7 after 24 h and 48 h compared to the untreated control (Figure 6a–d).

### 3.4. Examination of Tissue Biopsies

**Figure 7:**
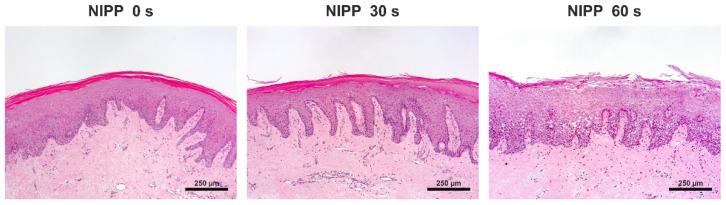
Effects of NIPP on gingival tissue biopsies at 24 h after NIPP treatment for 30 s and 60 s, as compared to the untreated group (NIPP 0 s). Hematoxylin and eosin (HE) staining, original magnification 10×. The scale bar represents 250 µm.

To evaluate the in vitro data, gingival tissue was treated with NIPP in vitro and analysed. For this purpose, tissue biopsies were treated with NIPP immediately after collection, incubated for 24 h, and examined histologically. Analysis of the sections showed that an application of 60 s NIPP led to damage of the superficial keratinocyte layer and slight dissolution of the suprabasal layers in the tissue (Figure 7).

**Figure 8:**
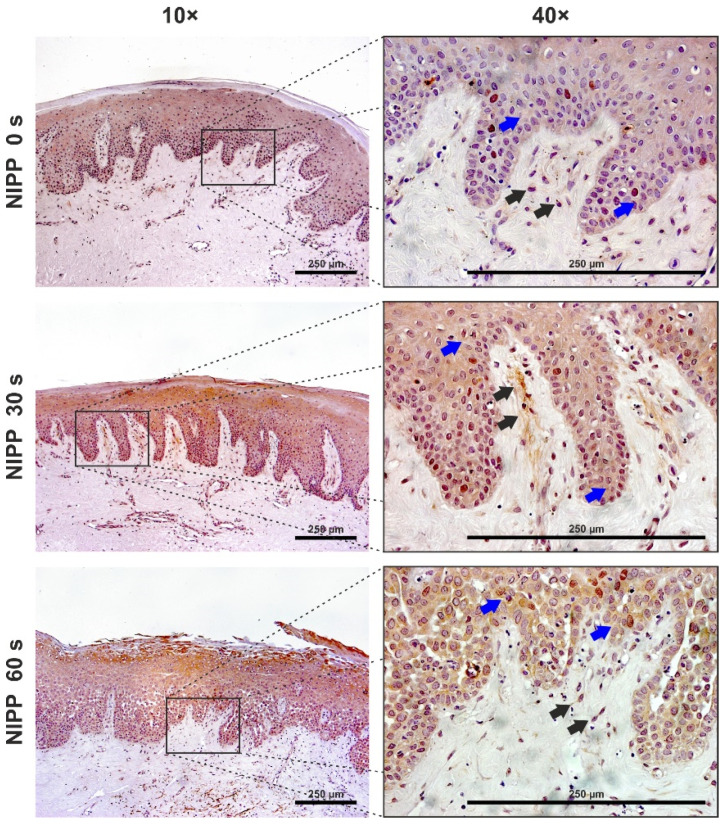
Influence of NIPP on gingival tissue biopsies at 24 h after NIPP treatment for 30 s and 60 s, as compared to the untreated group (NIPP 0 s). Ki67 staining, original magnification 10× and 40×. Blue arrows: positively stained HGK, black arrows: positively stained HGF. The scale bar represents 250 µm.

The expression of Ki67 could also be detected in the tissue biopsies. Since the tissues were stored in cell culture medium, the control group also showed Ki67 expression. However, 30 s NIPP application, in particular, caused an increase in Ki67 expression in the fibroblasts of the connective tissue and the basal layer of the keratinocytes. A 60 s NIPP application resulted in increased immunostaining of the superficial keratinocyte layers and reduced staining in basal layers. (Figure 8).

**Figure 9:**
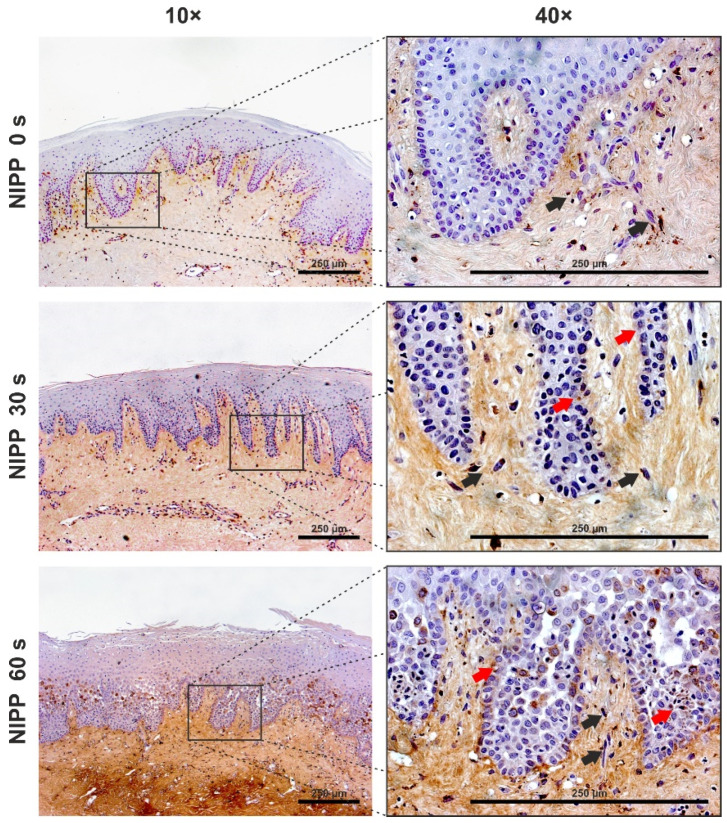
Influence of NIPP on gingival tissue biopsies at 24 h after NIPP treatment for 30 s and 60 s, as compared to the untreated group (NIPP 0 s). MMP1 staining, original magnification 10× and 40×. Red arrows: positively stained HGK, black arrows: positively stained HGF. The scale bar represents 250 µm.

Finally, we also investigated the expression of MMP1 in tissue biopsies. Here, in particular, the strong dose-dependent effects of NIPP could be demonstrated, which were especially evident in the subepithelial layer. Additionally, some keratinocytes were stained in the basal layer of epithelium. A 30 s NIPP treatment resulted in an increase in MMP1 staining throughout the whole lamina propria, and this was even more pronounced with a 60 s treatment (Figure 9).

## 4. Discussion

**Figure 10:**
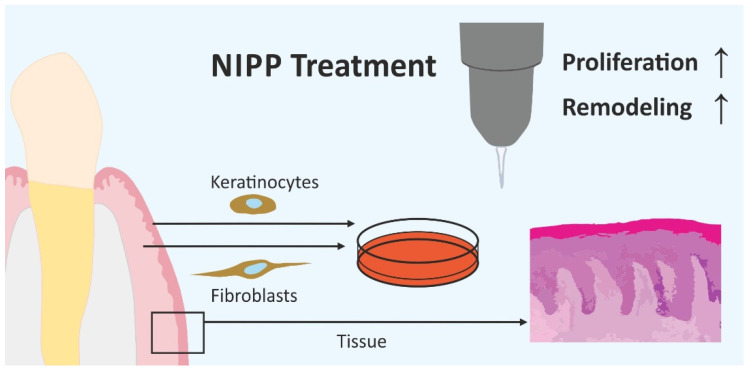
Markers of proliferation and remodeling were upregulated by NIPP in HGF, HGK, and tissue biopsies.

In the present study we have shown the proliferation-promoting effect of NIPP generated by an argon plasma device on gingival tissue concerning crucial markers regarding tissue regeneration and wound healing (Figure 10). In previous in vitro studies, we have shown similar effects of DBD-generated NIPP on hard tissue cells [28,29]. However, as the soft tissue is crucial for the healing of the underlying hard tissue without complications, the aim of this study was to investigate the effect on gingival cells and tissue.

First, we focused on mRNA expression of different molecules crucial for wound healing. For this reason, we examined the mRNA expression of *Ki67* and *PCNA*, which are well-established markers for the detection of proliferating cells [16] and additionally focused on Ki67 protein expression. In HGF cells, the proliferative effect is strongest with 30 s NIPP treatment and decreases with longer application times. In HGK, similar effects were observed, but Ki67 protein expression showed the same pattern as mRNA expression only after 48 h, which might be due to the time needed to transcribe the mRNA information into the protein.

The proliferation-promoting effect of NIPP has already been described in the literature [29,30,31,32,33,34,35,36]. In previous in vitro studies, we have shown an upregulation of *Ki67* and *PCNA* expression after 60 s of NIPP treatment at 24 h in PDL cells, gingival keratinocytes, and cementoblasts using a DBD [30,35,37]. Interestingly, after one day in HGK using a DBD, 60 s NIPP treatment revealed similar results in regard to *Ki67* mRNA expression, as we have shown in the present study, using an argon plasma jet. However, mRNA expression of *PCNA* after 60 s of NIPP treatment was higher using a plasma jet than using a DBD [37]. Further studies to systematically compare the two devices are needed to better understand the exact effect of NIPP on gingival cells. Since in the present study we demonstrated an effect on *Ki67* and *PCNA* for only 10 s of NIPP treatment after one day, even short applications seem to have an effect, which would facilitate clinical use. Other authors have shown this proliferative effect with as little as 3 s of NIPP treatment in vivo after 6 days, using the same device as we have used [32]. But different applications of NIPP have also been described in the literature: Park et al. (2019) showed an upregulation of PCNA protein expression in adipose tissue-derived stem cells after NIPP treatment of 50 s per h for 10 times at 24 h and 72 h, respectively [38]. For this reason, further studies are necessary to investigate different application times, such as 20 s, 40 s, and 50 s of NIPP treatment. It is also possible that the maximum of mRNA and protein expression is not at a treatment time of 30 s, but between 10 s and 30 s or 30 s and 60 s. Only further studies can illuminate this. In addition, the different effects of the various devices on the same cells should also be systematically examined.

It must also be considered that protein expression must also have an effect on cell number. However, in the previous in vitro studies mentioned above, we were also able to show that the NIPP-regulated upregulation of *Ki67* and *PCNA* correlates with an increasing cell number: Thus, a significant increase in cell number was shown after 24 h in osteoblast-like cells [29]. However, a systematic investigation of the cell number with regard to different time points should also be the target of further studies with HGF and HGK.

Furthermore, the influence of NIPP on other components of in vitro cell regeneration, such as the metabolic activity of the cells or cell migration, has been described in the literature: Cui et al. have described increased cell migration and higher levels of angiogenic growth factors in human keratinocytes treated with LTP for 30 s, 60 s and 180 s using a DBD plasma device [36]. Liu et al. used MTT to demonstrate an increase in cell viability in mouse fibroblasts in vitro after 48 h when exposed to an argon plasma jet at 0.5 L/min from 0–30 s: They showed a peak at 15 s, which might be due to the different device or a different, not specified distance to the cells [39]. Nevertheless, it must be taken into account that different cell types and cells from different tissues can show different cell responses to the same stimuli—as already shown for HGF and HGK.

Further on, we focused on COL1A1 and MMP1, which are both important regulators of early wound healing [40,41]. COL1A1 is mainly produced by fibroblasts rather than keratinocytes, although keratinocytes are able to control the production of COL1A1 in fibroblasts [4]. However, we decided to also investigate the mRNA expression of *COL1A1* in keratinocytes, as platelet-released growth factors (e.g., platelet-rich fibrin) have been described to stimulate *COL1A1* mRNA expression in keratinocytes [42]. Interestingly, *COL1A1* mRNA regulation was not substantially affected by plasma in either HGF or HGK. In previous in vitro studies, we found an upregulation of *COL1A1* mRNA after NIPP treatment of osteoblasts, PDL cells, and cementoblasts using a DBD [29,30,35]. In addition, dermal and gingival fibroblasts have both been described to show increased mRNA expression of *COL1A1* after NIPP application for 1, 3 and 5 min, and 1, 2, and 4 min in vitro [43,44]. We can only speculate about the reasons for the differences in the literature, but on the one hand cells seem to react differently to NIPP influence, while on the other hand all the authors have used different NIPP devices and different experimental settings: Osteoblasts, PDL cells and cementoblasts were stimulated by a DBD [29,30,35]; dermal fibroblasts were stimulated by a non-commercial microwave plasma generator [43], and HGF were stimulated by an experimental plasma jet supplied by compressed air at 1 L/min [44]. Since our device operates with argon gas at higher gas flow (4 L/min), additional research is needed to further decipher the differences of the various devices on gingival cells.

Furthermore, MMP1 was upregulated by NIPP both at the mRNA level and at the protein level in the cell culture supernatant in a dose-dependent manner. We have already been able to demonstrate the promoting effect of NIPP on *MMP1* in cells of the periodontal ligament and in hard tissue cells using a DBD in vitro [29,30]. Therefore, we can assume that the effect of NIPP on the regulation of *MMP1* is independent of the technology used to generate NIPP. However, as elevated MMP1 levels have also been associated with periodontitis [45], it must be ruled out that NIPP is not harmful to gingival cells and tissue. However, since we have shown that short treatment times lead to early MMP1 upregulation in HGK and HGF without affecting the regulation of apoptotic markers, this seems to be ruled out. Other methods that promote wound healing also have an effect on increased MMP1 secretion, such as the use of platelet-rich plasma [46].

Longer NIPP applications, such as 60 s and especially 90 s treatment, are associated with upregulation of *CASP3* and *CASP9* mRNA. Interestingly, this does not result in higher caspase-3/7 activity. It is possible that NIPP seems to irritate the cells but prevents the cells from actually becoming apoptotic. This underlines the non-invasive effect of NIPP.

Apoptotic effects of NIPP have been abundantly described in the literature, especially for malignant cells [47,48,49]. In non-malignant cells, the antiproliferative effect is evident when NIPP is applied for a long time: In HaCaT keratinocytes, 180 s NIPP treatments have been described as apoptotic after 24 h [50]. Additionally, Weiss et al. have shown a significant decrease in the cell number of fibroblasts after 72 and 120 h when treated for 120 s [51]. Further studies are needed here to determine whether the longer NIPP treatment in HGK and HGF is also reflected in a decrease in cell number. It is possible that the direct NIPP effects regarding apoptosis might already be detectable a few hours after NIPP application. However, in previous studies, we also observed downregulation of mRNA regulation of apoptotic genes and an anti-apoptotic effect in PDL cells and MG63 cells by NIPP [29,30]. However, one reason for the difference could be the device—in those studies an ambient air-controlled DBD was used, whereas in the present study we have used an argon plasma jet. Weiss et al. and Schmidt at al. have also used plasma jets [50,51]. Interestingly, in the literature overall harmful effects of NIPP jets on gingival cells have been described: Lee et al. demonstrate a harmful effect of compressed-air plasma (3 L/min) on HGF viability in vitro [52]. However, in this study, in contrast to our study design, the cells were not covered with medium during the NIPP treatment. Regarding our results, it must also be borne in mind that distance and continuous movement play a role. Thus, we were also able to induce apoptotic effects on HGF and HGK with our NIPP device at closer distances without continuous movement of the jet (data not shown). In addition, it must also be taken into account that the gas flow of 4 L/min might dry out the medium, even though we did not observe any significant difference in the amount of medium after NIPP treatment compared to the untreated control when collecting the cell culture supernatants for the ELISA experiments. However, further in vitro studies between medium-covered and non-covered cells are necessary to fully unravel the effects of gas flow and NIPP on the cells. Additionally, as there is no gas flow with DBD devices, a systematic investigation of the influence of these devices on gingival cells would help to further unravel the effect of NIPP in dentistry.

In the present study, we also focused on the examination of tissue biopsies where the monolayer results could be confirmed. Interestingly, we observed a slight injury of the superficial keratinocyte layer after 60 s NIPP, which could be an effect of the direct treatment. The difference from our in vitro experiments could be due to the fact that the cells on the surface already become apoptotic immediately after treatment or that the damage is attributed to a different mechanism. Further research on apoptosis is necessary to better understand this difference. Jablonowski et al. have, however, described these side effects of NIPP generated by a medical argon plasma device in an in vivo study: 10 s and 60 s NIPP cause focal mucosal erosion with superficial ulceration after 1 d in mice, but heal within a week without complications [53]. Furthermore, this superficial lesion was considered non-carcinogenic [54]. It seems that these superficial injuries do not contradict the clinical use of NIPP: Clinical studies have shown wound-healing effects on gingival wound healing regarding application times of 60 s using a plasma jet and 120 s using a DBD [26,27]. Since clinical healing of the gingiva was observed even with prolonged applications of NIPP, the observed superficial lesions do not seem to be relevant. This is confirmed by our results showing expression of Ki67 and MMP1 protein in the tissue layers beneath the epithelium. These results correspond to the results of other authors who have found NIPP effects at tissue depths of up to 2 mm [55]. However, further in vivo and in vitro studies are necessary to better understand the direct effect of NIPP on gingival wound healing.

Nevertheless, other methods of applying NIPP would be possible in dentistry, which might achieve a similar effect: Lou et al. observed an increase in Ki67 protein expression after 24 h using NIPP activated medium [33]. This possibility of applying multibiological functions to the fluid through NIPP treatment would greatly simplify its application in dentistry, allowing it to be used as a mouth rinse [56].

Whatever the method of applying NIPP, the use of NIPP is a cost-effective and time-saving method to improve gingival wound healing. In particular, patients with impaired wound healing, such as diabetics or oncological patients, could benefit. A reduction of these wound-healing disorders would not only have a psychological benefit for the patients but would also help to save costs in the health care system [57,58].

However, this study has some limitations: For protein analysis, we focused only on the selected markers Ki67 and MMP1. Since *PCNA* mRNA was also strongly regulated by NIPP, the analysis of protein levels after NIPP treatment would also have been useful. However, we followed the publication of Bologna-Molina et al., who identified both markers as similar proliferation markers, but gave Ki67 a higher specificity regarding ameloblastic cells [16]. Further studies comparing Ki67 and PCNA directly in HGF and HGK could be interesting to further decipher the NIPP effect on cell proliferation in gingival cells. Additionally, we used tissue biopsies to study NIPP effects. Since this tissue is only nourished by the cell culture medium, only marginal conclusions can be drawn about the biological effect within a living organism. This had an impact on the Ki67 staining, as the cells in the tissue were constantly proliferating due to storage in medium, requiring us to adjust the antibody dilution to visualise differences between the groups. For this reason, fibroblast staining within the tissue is low. However, the penetration depth of NIPP has already been demonstrated in the literature [59]. Additionally, we studied tissue biopsies at one day because of the risk of tissue degradation in the humified incubator, regarding a longer treatment time. For this reason, further in vivo experiments with a focus on intraoral wound healing are necessary to further understand the effect of NIPP. However, the aim of the present study was to use human samples, which is more representative of the patients who will be treated with NIPP. Furthermore, it must be borne in mind that commercially available cells were used for the monolayer experiments. Although we were able to confirm cell culture results in the tissue model with regard to MMP1 and Ki67, primary HGF and primary HGK may show different results of gene and protein expression. Further studies should therefore investigate this point in more detail.

## 5. Conclusions

Within the limits of our in vitro study, we could demonstrate that NIPP generated by a medical argon plasma device promotes wound healing-associated processes in human gingival cells and tissue. Although the exact effects of different NIPP devices on the gingiva are not yet known, it seems that argon-based plasma jets offer promising prospects for patients suffering from delayed wound healing and, last but not least, offer all patients the prospect of not being limited too long after surgical interventions.

## Data Availability

Not applicable.
